# Antitrypanosomal Effects of* Zanthoxylum zanthoxyloides* (Lam.) Zepern. & Timler Extracts on African Trypanosomes

**DOI:** 10.1155/2019/1730452

**Published:** 2019-07-04

**Authors:** Aboagye Kwarteng Dofuor, Georgina Isabella Djameh, Frederick Ayertey, Peter Bolah, Michael Amoa-Bosompem, Kwaku Kyeremeh, Laud Kenneth Okine, Theresa Manful Gwira, Mitsuko Ohashi

**Affiliations:** ^1^West African Center for Cell Biology of Infectious Pathogens, University of Ghana, Legon, Ghana; ^2^Department of Biochemistry, Cell and Molecular Biology, University of Ghana, Legon, Ghana; ^3^Department of Parasitology, Noguchi Memorial Institute for Medical Research, University of Ghana, Legon, Ghana; ^4^Center for Plant Medicine Research, Mampong-Akuapem, Ghana; ^5^Department of Chemistry, University of Ghana, Legon, Ghana; ^6^Department of Environmental Parasitology, Tokyo Medical and Dental University, Tokyo, Japan

## Abstract

African trypanosomiasis is a disease caused by the parasitic protozoa of the* Trypanosoma* genus. Despite several efforts at chemotherapeutic interventions, the disease poses serious health and economic concerns to humans and livestock of many sub-Saharan African countries.* Zanthoxylum zanthoxyloides *(Lam.) Zepern. & Timler (*Z. zanthoxyloides *LZT) is a plant species of important phytochemical and pharmacological relevance in the subtropical zones of the African continent. However, the mechanisms of its antitrypanosomal effects in African trypanosomes remain to be elucidated. The aim of the study was to determine the* in vitro *effects and mechanisms of action of* Z. zanthoxyloides *LZT (root) fractions against* Trypanosoma brucei*.* T. brucei *(GUTat 3.1 strain),* L. donovani *(D10 strain),* P. falciparum *(3D 7 strain), Jurkat cells, and Chang liver cells were cultivated* in vitro *to the log phase in their respective media at 37°C. Crude extracts and fractions were prepared from air-dried pulverized plant material of* Z. zanthoxyloides *LZT (root) using the modified Kupchan method of solvent partitioning. Half-maximal inhibitory concentrations (IC_50_) were determined through the alamar blue cell viability assay. Effects of fractions on cell death and cell cycle of* T. brucei* were determined using flow cytometry. Fluorescence microscopy was used to investigate the effects of fractions on the morphology and distribution of* T. brucei*. Antitrypanosomal compounds of fractions were characterized using high-performance liquid chromatography (HPLC) and attenuated total reflectance infrared (ATR-IR) spectroscopy. Methanol, butanol, and dichloromethane fractions were selectively active against* T. brucei *with respective IC_50_ values of 3.89, 4.02, and 5.70 *μ*g/ml. Moreover, methanol, butanol, and dichloromethane fractions significantly induced apoptosis-like cell death with remarkable alteration in the cell cycle of* T. brucei*. Furthermore, dichloromethane and methanol fractions altered the morphology, induced aggregation, and altered the ratio of nuclei to kinetoplasts in the parasite. The HPLC chromatograms and ATR-IR spectra of the active fractions suggested the presence of aromatic hydrocarbons with hydroxyl, carbonyl, amine, or amide functional groups. The results suggest that* Z. zanthoxyloides *LZT have potential chemotherapeutic effects on African trypanosomes with implications for novel therapeutic interventions in African trypanosomiasis.

## 1. Introduction

African trypanosomiasis (AT) is a tsetse-transmitted disease of humans and livestock caused by the parasitic protozoan genus* Trypanosoma*. Human African trypanosomiasis (HAT) and animal African trypanosomiasis (AAT) form the two main types of AT [1, 2]. An estimated 70 million population in about 36 countries of sub-Saharan Africa may be at risk of HAT while AAT threatens the lives of millions of cattle every year [1, 2]. HAT is caused by two subspecies of* Trypanosoma brucei *(*T. brucei*) that infect either humans (*T. b. gambiense*) or animals (*T. b. rhodesiense*) [1], while AAT threatens the lives of several million herds of cattle every year thereby requiring new approaches of combating the disease [2].

The prospect of vaccine development for AT is beset with various challenges partly due to the parasite's immune evasion strategies that include antigenic variation of variant surface glycoproteins, trypanolytic factor defense, and modulation of the immune system [3]. Hence, the most efficient and economically viable option is chemotherapy. AAT may be treated with isometamidium, homidium, diminazine, pyritidium, and quinapyramine, while HAT is usually treated with pentamidine, eflornithine, nifurtimox, melarsoprol, and suramin depending on the stage of the disease [4, 5]. However, challenges such as drug resistance, undesirable side effects, and difficulty in regimen application are usually associated with trypanosomiasis chemotherapy [5–10]. In view of the fact that about two-thirds of the world population depends on traditional medical remedies as a result of the limited affordability and availability of pharmaceutical products [11], a better understanding of such antitrypanosomal effects is required if novel drugs that can circumvent the challenges associated with the currently available antitrypanosomal drugs are to be developed.

*Zanthoxylum *is a genus of deciduous and evergreen trees and shrubs which comprises at least 500 species [12]. It is a widely distributed plant genus native to most temperate and subtropical zones of Africa, Asia, North America, South America, and Australia. Traditionally, the leaves, stem, and roots of* Zanthoxylum *are used in medicinal preparations for the treatment of diseases such as colic, toothache, stomachache, and oral infections [13]. The medicinal values of* Zanthoxylum *may be attributed to various pharmacological properties, namely, antimicrobial, insecticidal, anti-inflammatory, antioxidant, antiparasitic, antitumor, antihelmintic, antinociceptive, and antiviral activities [12]. For instance, the aqueous extract of the root of* Zanthoxylum zanthoxyloides *(Lam.) Zepern. & Timler (*Z. zanthoxyloides *LZT), one of the most important species on the African continent, was reported to exhibit analgesic effects probably via the inhibition of prostaglandin [14]. Aqueous and ethanolic extracts from the leaves of the same species also exhibited antihelmintic activities against* Ascaris lumbricoides*,* Haemonchus contortus*, and* Trichostrongylus colubriformis *[15, 16].

*Zanthoxylum *is endowed with a vast array of diverse phytochemicals which may largely be responsible for its pharmacological effects. Canthine-6-one alkaloids isolated from* Z. chiloperone *Mart. ex Engl contributed towards the significant reduction of parasitemia and serological response after treatment in mice [17–20]. Moreover,* Z. zanthoxyloides *LZT is known to possess pharmacologically useful phytochemicals such as acridones, citronellol, and divanilloylquinic acids [21–23], which might have contributed to the trypanostatic effect observed after treatment in mice [24, 25]. However, the mechanisms of antitrypanosomal activity were not investigated in any of these studies.

Determination of the effects and mechanisms of antitrypanosomal action of* Zanthoxylum *may foster the development of novel drugs that can circumvent the challenges associated with the currently available antitrypanosomal drugs. The aim of this study was thus to determine the active fractions of the Ghanaian species of* Z. zanthoxyloides *LZT (root) as well as to determine their effects and mechanisms of action using a panel of cell biological approaches.* Z. zanthoxyloides *LZT (root) significantly induced apoptosis-like cell death and resulted in a significant alteration of the cell cycle and morphology of* T. brucei*. Our results suggest that* Z. zanthoxyloides *LZT (root) has promising antitrypanosomal effects with implications for potential therapeutic interventions towards AT.

## 2. Methods

### 2.1. Culture of Parasites and Human Cell Lines

Blood stream forms of the subspecies* T. b. brucei *(GUTat 3.1 strain) and Jurkat cells (acute lymphoblastic leukemia cells) were cultivated* in vitro *to the log phase using Hirum's Modified Iscove's Medium (HMI9, Thermo Fisher Scientific) with 10% fetal bovine serum (FBS) (Thermo Fisher Scientific) at 5% CO_2_ and 37°C. Chang liver (HeLa derivative) cell lines were cultivated* in vitro *to the log phase using Minimum Essential Medium (MEM, Thermo Fisher Scientific) with 10% FBS at 5% CO_2_ and 37°C.* L. donovani *(D10 strain) was cultivated* in vitro *to the log phase using Medium 199 (M199, Thermo Fisher Scientific) with 10% FBS at 5% CO_2_ and 37°C.* P. falciparum *(3D7 strain) was cultivated* in vitro *to the log phase using the Roswell Park Memorial Institute Medium (RPMI 1640, Gibco) in culture flasks containing packed red blood cells suspended at 4% hematocrit.* Plasmodium *cultures were maintained with a gas mixture (2% O_2_, 5% CO_2_ and 93% N_2_) at 37°C.

### 2.2. Crude Extraction and Fractionation of Plant

The present study focused on the Ghanaian plant species* Z. zanthoxyloides *LZT. The plant was collected from an arboretum and the environs of the Center for Plant Medicine Research (CPMR), Mampong-Akuapem, Ghana. It was authenticated by Herone Blagogee, a senior botanist at the Plant Development Department of CPMR and voucher specimen number CPMR 4120/4121/4122 assigned and lodged at the herbarium of CPMR. Crude extracts and fractions were prepared from the air-dried pulverized plant material by extraction with dichloromethane and methanol followed by the modified Kupchan method [26] of solvent extraction. Briefly, the pulverized air-dried plant material was soaked in dichloromethane and left in the cold to percolate for one week. The dichloromethane extracts were decanted and filtered through a mixture of cotton and glass wool. The plant material was then soaked in methanol for one week and the methanol extract was decanted and filtered. The methanol and dichloromethane extracts were combined and dried under vacuum with a Heidolph Rotavap at 40°C and 1 atm pressure to give the total crude extract (TCE). The TCEs were suspended in water and extracted three times with dichloromethane. The remaining aqueous layer was then extracted once with* sec*-butanol and the butanol fraction was dried under vacuum to give the WB fraction. The dichloromethane layer was dried under vacuum and the extract was suspended in a 1:9 mixture of water and methanol. This fraction was then extracted three times with hexane after which the hexane layer was dried under vacuum to give an FH fraction. The remaining 1:9 mixture of water and methanol layer was phase adjusted to a 1:1 mixture, extracted three times with dichloromethane, and dried under vacuum to give an FD fraction. The 1:1 water methanol layer was also dried under vacuum to give the FM fraction.

### 2.3. Chromatography and Spectroscopy Analysis

Mid-infrared (IR) spectroscopy was carried out by the Universal Attenuated Total Reflectance (UATR) spectrometer with the following instrument (PerkinElmer) specifications: spectrum version=10.03.09; model=spectrum 2; serial number=94133; number of scans=24; resolution=4. High-performance liquid chromatography (HPLC) was performed using the following instrument (Agilent) specification: pump=G4289A, detector=UV G1314, data software=chemstation enterprise view, column=COSMOSIL Cholester (4.6 x 250 mm) (Nacalai Tesque INC), chromatography interface=Agilent 1120 compact LC. The chromatographic conditions used included: column temperature=30°C; injection volume= 10 *μ*l; flow rate= 1 ml/min; wavelength=254 nm; solvent system=acetonitrile: 0.1% V/V formic acid (100:0, 10 mins; 90:10, 5 mins; 80:20, 50 mins; 70:30, 5 mins; 60:40, 5 mins; 50:50, 5 mins).

### 2.4. Cell Viability Analysis

Determination of cell viability involved colorimetric analysis through the use of resazurin (alamar blue) [27].* T. brucei, L. donovani, P. falciparum, *and Jurkat cells were seeded at a density of 1.5x10^5^ cells/ml on 96-well plates with the fractions and incubated for 24 hours. Alamar blue dye (10% V/V) was added to wells and incubated for another 24 hours. Chang liver (HeLa derivative) cells were plated at a density of 1.5x10^5^ cells/ml for 24 hours to allow for sufficient adherence to plates, before fractions were added to cells and incubated for another 24 hours. Alamar blue dye (10% V/V) was then added to wells and incubated for another 72 hours to allow for a complete color change. Experiments were run in triplicate. Spectrophotometric absorbance was read at a wavelength of 540 nm using a reference wavelength of 595 nm.

### 2.5. Analysis of Apoptosis

Flow cytometry-based detection of apoptosis-like cell death using annexin-V and 7-amino actinomycin-D was employed [28]. Cells were seeded at a density of 1.5x10^5^ cells/ml on a 96-well plate in a twofold dilution of fractions and incubated for 24 hours. Nexin reagent containing annexin-V and 7-amino actinomycin-D (EMD, Millipore) was added to each well in a volumetric ratio of 1:1. Plates were incubated in darkness for 20 mins with gentle shaking. Each experiment was run in duplicate. Dot plots were recorded with the Guava easyCyte HT flow cytometer.

### 2.6. Analysis of Cell Cycle

The cell cycle assay was based on a univariate analysis of DNA content upon staining with propidium iodide [29]. Cells were seeded at a density of 3.0x10^5^ cells/ml in 25cm^2^ culture dish with or without fractions for 24 hours and centrifuged at 1700 rpm for 10 mins. Cell pellets were suspended in 1.5 ml of 1x phosphate-buffered saline (PBS) and vortexed well. A 3.5 ml of absolute ethanol was added (final concentration of 70%) to fix cells at -20°C for 1 hour. Cells were centrifuged at 1700 rpm for 10 mins. Cell pellets were suspended with 200 *μ*l of guava cell cycle reagent that contained propidium iodide (EMD, Millipore). Suspended cells were added to wells containing the same volume of fresh guava cell cycle reagent. Cells were incubated for 30 mins in darkness at room temperature. Distribution of cells at distinct cell cycle phases was measured with the BD LSFortessa X-20 flow cytometer.

### 2.7. Fluorescence Microscopy

Cells were cultured with or without fractions for 24 hours and centrifuged at 1700 rpm for 10 mins. Cell pellets were suspended in 375 *μ*l of PBS and vortexed. A 125 *μ*l of 16% paraformaldehyde (final concentration of 4%) was added to pellets, incubated for 5 mins at room temperature, and spun at 1700 rpm for 5 mins. Cell pellets were suspended in 500 *μ*l of PBS and 50 *μ*l were put on a glass slide. Cells on the glass slide were incubated at room temperature for 1 hour. They were rinsed once in PBS for 5 mins, followed by a second rinse in PBST (PBS with 0.1% Triton X) for 15 mins. They were then incubated with 4', 6-diamidino-2-phenylindole (DAPI) (5*μ*g/ml in PBS) for 10 mins in the dark at room temperature. Cells were washed twice in PBS for 5 mins each, followed by a second single wash in PBST for 5 mins. Cells were mounted with 90% glycerol in PBS using a cover slip, sealed with manicure, and observed with the Olympus DP72 reflected fluorescence microscope. Data was analysed with the CellSens standard imaging software and Adobe Photoshop CS6.

### 2.8. Statistical Analysis

Data from cell viability assay was analysed with Graphpad Prism (version 5) and Microsoft Excel. The half-maximal inhibitory concentration (IC_50_) was calculated as the concentration that caused a 50% reduction in cell viability. IC_50_ were calculated from a nonlinear regression model as statistically appropriate. Dot plots from cell death and cell cycle assays were analysed with the guavaSoft 2.1 and BD FACSDiva 8.0.1, respectively. Histograms for cell cycle were generated with FlowJo V10. Statistical analysis of percentage counts was carried out with Graphpad Prism version 5 using the unpaired t-test.* P* values ≤0.05 were considered to be significant.

## 3. Results

### 3.1. *Z. zanthoxyloides *LZT (Root) Possesses Selective Antitrypanosomal Activity

**Figure 1:**
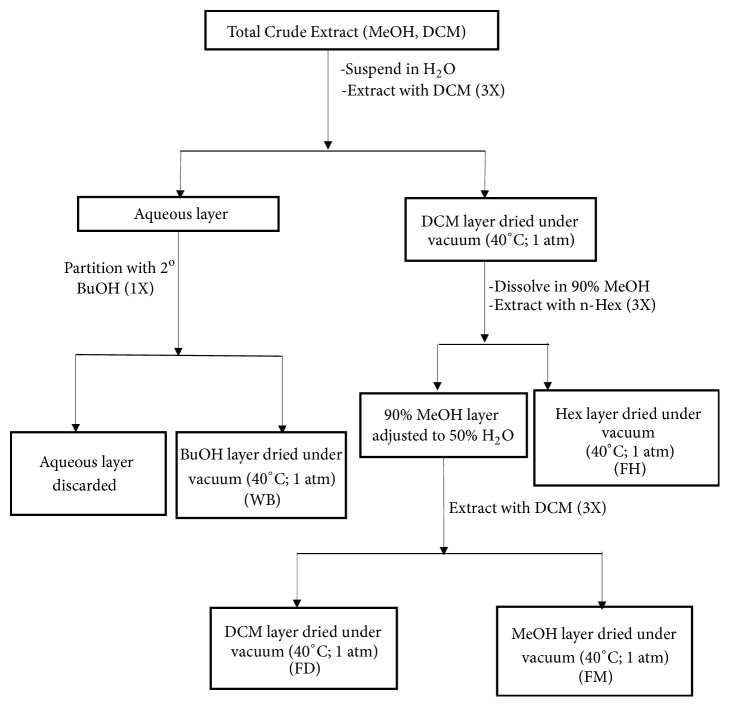
*Schematic for modified Kupchan method of liquid-liquid extraction.* The plant material was macerated in dichloromethane and the extracts were decanted and filtered. The plant material was then soaked in methanol for one week and the methanol extract was decanted and filtered. The methanol and dichloromethane extracts were combined and dried under vacuum to give the total crude extract (TCE). Extraction with solvents of varying polarities then gave rise to 4 fractions: butanol fraction (WB), hexane fraction (FH), dichloromethane fraction (FD), and methanol fraction (FM). ZR=*Z. zanthoxyloides *LZT root; MeOH=methanol; DCM=dichloromethane; BuOH=butanol; Hex=n-hexane.

The antiparasitic activity of the total crude extracts of different parts of* Z. zanthoxyloides *LZT (root, stem bark, and leaves) prepared by cold maceration in dichloromethane and methanol (Figure 1) was determined in a 48-hour alamar blue cell viability assay against three protozoan parasites:* T. brucei, Leishmania donovani (L. donovani), *and* Plasmodium falciparum (P. falciparum) *(Table 1). The assay also included Jurkat (acute lymphoblastic leukemia cells) to investigate potential selectivity profiles of* Z. zanthoxyloides *LZT in the parasites. Respectively, the root, stem bark, and leaves of* Z. zanthoxyloides *LZT exhibited different levels of activity and selectivity towards* T. brucei *(IC_50_=3.41 *μ*g/ml,* P*<0.001, SI=72.48; IC_50_=39.43 *μ*g/ml,* P*<0.01, SI=6.27; IC_50_=27.73 *μ*g/ml,* P*<0.01, SI=3.46) (Table 1). Moreover, while the root exhibited the strongest activity and selectivity (IC_50_=3.41 *μ*g/ml,* P*<0.001, SI=72.48) against* T. brucei*, the activity of the stem bark (IC_50_=13.50 *μ*g/ml,* P*<0.01, SI=18.30) was the strongest against* L. donovani *(Table 1). The activity and selectivity profiles against* P. falciparum *were the weakest in all the plant parts.

Due to the promising antitrypanosomal activity and selectivity profiles of the root extract of* Z. zanthoxyloides, *LZT different solvent fractions were prepared from the root of the same plant material to characterize the chemical properties using spectroscopy and chromatography. This characterization may also provide support for the presence of phytochemicals as being responsible for the observed antitrypanosomal activities. ZRFH (*Z. zanthoxyloides *LZT, root, hexane fraction), ZRFD (*Z. zanthoxyloides *LZT, root, dichloromethane fraction), ZRFM (*Z. zanthoxyloides *LZT, root, methanol fraction), and ZRWB (*Z. zanthoxyloides *LZT, root, water butanol fraction) were prepared from* Z. zanthoxyloides *LZT (root) using the Kupchan method of solvent extraction (Figure 1). The crude extract was prepared through a stepwise maceration in absolute methanol and absolute dichloromethane for 1 week each. The two solvent extracts were combined and dried under vacuum to form the total crude extract from which ZRFH, ZRFD, ZRWB, and ZRFM were prepared as outlined diagrammatically (Figure 1).

Antitrypanosomal compounds in each Kupchan fraction were then characterized using attenuated total reflectance infrared (ATR-IR) spectroscopy (Table 2, Additional file 1) and analytical high-performance liquid chromatography (HPLC) (Table 3, Additional file 2). All the fractions exhibited absorbance intensities within a range of wavenumbers suggesting the presence of carbonyl (1950-1450 cm^−1^) and aromatic (900-400 cm^−1^) groups (Table 2). The IR spectra were also suggestive of the presence of hydroxyl, amine, or amide groups in the fractions (3700-2500 cm^−1^) (Table 2, Additional file 1). The methanol and dichloromethane fractions were particularly similar in their IR spectra, although they differed in the exact transmittance intensities at the respective wavenumbers. HPLC was performed using a gradient solvent system of absolute acetonitrile and 0.1% V/V formic acid. The absorbance of individual compounds was recorded with an ultraviolet detector system at 254 nm. The compounds in all the fractions separated within a range of minimum and maximum retention times of approximately 2 and 15 mins respectively (Table 3). The major compounds in hexane, butanol, dichloromethane, and methanol fractions were separated at retention times of 3.746, 3.732, 3.743, and 3.745 mins, respectively (Table 3). Several compounds of minor quantities that may have contributed to the selective antitrypanosomal activities of* Z. zanthoxyloides *LZT (root) were also detected at varying retention times in all the fractions (Table 3, Additional file 2).

**Figure 2:**
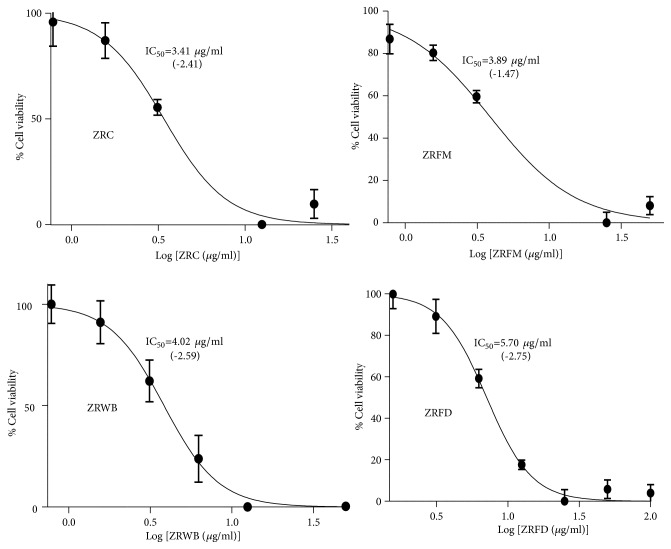
*Dose-response curves of selected fractions.* The best-fit inverted logistic dose-response curves and corresponding IC_50_s were modelled from three distinct alamar blue assays. The crude extract and its fractions have Hill slopes less than -1 (shown in parentheses). ZR=*Z. zanthoxyloides *LZT, root; ZRC=crude extract of ZR; FD=Dichloromethane fraction; FM=methanol fraction; WB= butanol fraction.

The fractions were then tested for their antitrypanosomal activities in a 48-hour alamar blue cell viability assay (Table 4). The assay also included Jurkat and Chang liver cells (HeLa derivatives) to investigate selectivity profiles of the promising fractions. ZRFM, ZRWB, and ZRFD stood out as the most promising antitrypanosomals with respective IC_50_ values of 3.89, 4.02, and 5.70 *μ*g/ml (*P*<0.001) (Table 4). Generally, fractions were more selective to the parasites as compared to Jurkat or Chang liver cells, albeit the selectivity was higher in the latter. ZRFD and ZRWB showed relatively high toxicity against Jurkat cells with respective selectivity indices (SI) of 9.13 and 6.29 while ZRFM, ZRFD, and ZRWB displayed low toxicity profiles towards Chang liver cells with SI of 24.12, 15.78, and 93.43, respectively (Table 4). Moreover, ZRFD, ZRFM, and ZRWB exhibited inverted sigmoidal dose-response curves with Hill coefficients less than -1 (Figure 2), which might be an indication of positively cooperative binding from a mechanistic point of view [30]. The inverted Hill coefficient was highest and lowest for ZRFD (-2.75) and ZRFM, respectively (-1.47) (Figure 2).

### 3.2. *Z. zanthoxyloides *LZT (Root) Induces Apoptosis-Like Cell Death and Cell Cycle Changes in* T. brucei*

**Figure 3:**
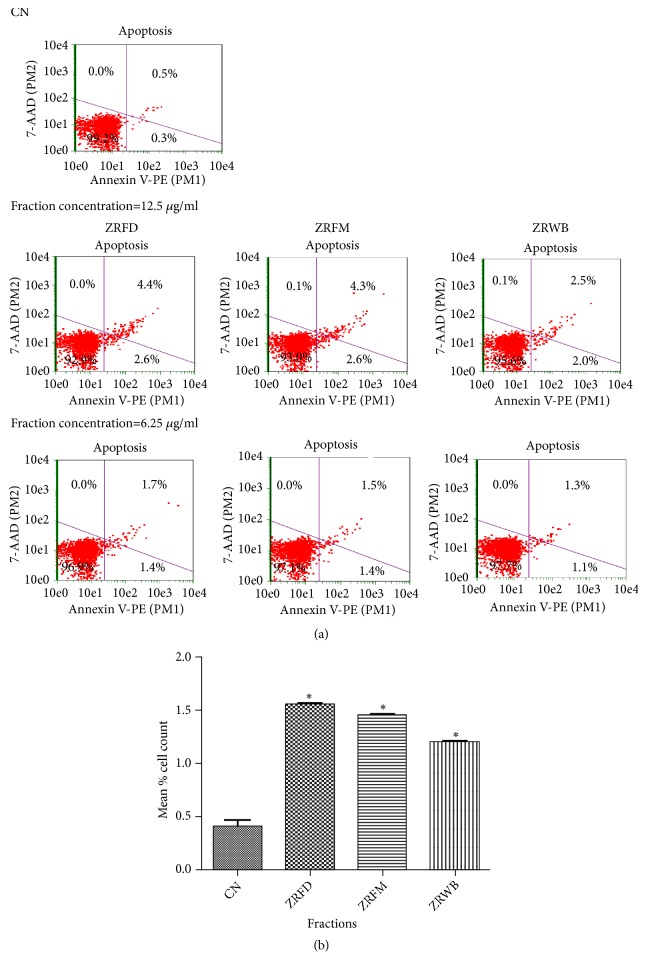
*Effect of selected fractions on induction of apoptosis in T. brucei. *(a) The assay measures the binding between phycoerythrin-bound annexin-V protein (annexin V-PE, X-axis) and phosphatidyl serine during either early apoptosis (lower right quadrant) or late apoptosis (upper right quadrant). It also captures the binding between 7-amino-actinomycin-D (7-AAD, Y-axis) and fragmented DNA during either late apoptosis (upper right quadrant) or necrosis (upper left quadrant). At lower left quadrant, there is predominance of viable cells that are negative for both annexin V-PE and 7-AAD. (b) P values were calculated from 3 distinct counts (n=3) [P:< 0.05(*∗*)]. Error bars originate from mean percentage count ± standard deviation of the mean (Mean ± SD). Fractions were investigated at the IC_50_ values. ZR=*Z. zanthoxyloides *LZT, root; WB=butanol fraction; FD=Dichloromethane fraction; FM=Methanol fraction; CN=Negative control.

Exploration of antitrypanosomal effects in the context of mechanisms of cell death and cell cycle can aid in the development of antitrypanosomal drugs. In order to determine effects on cell death, parasites challenged with fractions for 24 hours were used in a cell death assay (Figures 3(a) and 3(b)). The assay explored the interaction between phycoerythrin-bound annexin-V protein and phosphatidyl serine released from the integral side of the plasma membrane to the periphery during either early apoptosis or late apoptosis, as well as the binding between 7-amino-actinomycin-D (7-AAD) and the fragmented DNA during either late apoptosis or necrosis [28]. Doubling the concentration of individual fractions caused a corresponding increase in induction of apoptosis-like cell death. At the IC_50_, ZRFD, ZRFM, and ZRWB caused a significant induction of apoptosis-like cell death from 0.40% in the untreated control to 1.55%, 1.45%, and 1.20% of cells, respectively (*P*<0.05) (Figures 3(a) and 3(b)).

**Figure 4:**
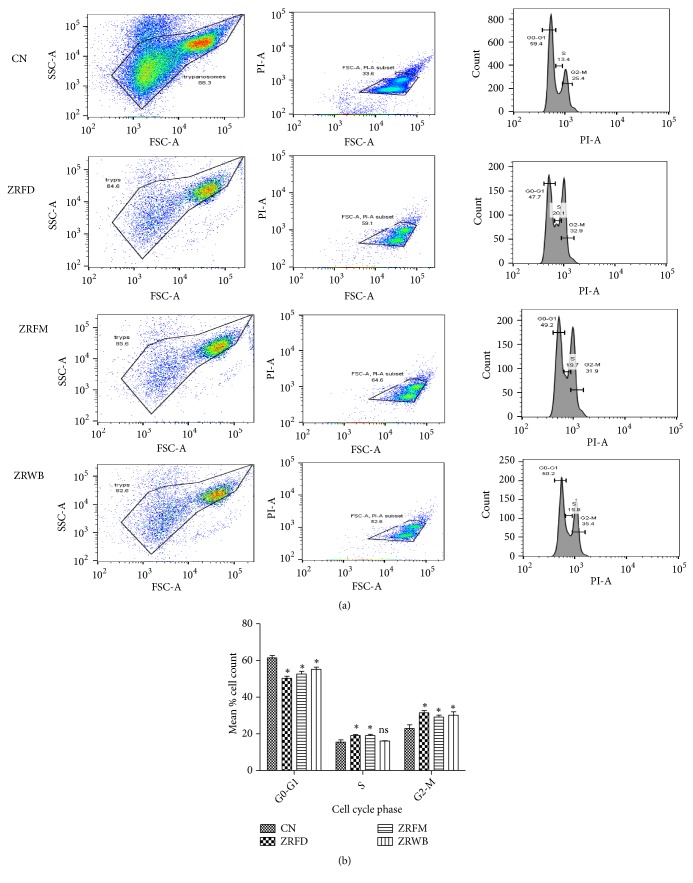
*Effect of selected fractions on cell cycle of T. brucei*. (a) Dot distribution of first column represents plot of side scatter versus forward scatter; second column compares intensity of propidium iodide with forward scatter; third column compares population count of parasites with intensity of propidium iodide. Three phases of the parasite are captured in the histograms: G0-G1, first peak; S, middle trough; G2-M, second peak. Fractions were investigated at the IC_50_ values. (b) P values were calculated from 4 distinct counts (n=4): (*∗P* < 0.05). Error bars originate from mean percentage count ± standard deviation of the mean (Mean ± SD). ZR=*Z. zanthoxyloides *LZT, root; WB=butanol fraction; FD=Dichloromethane fraction; FM=Methanol fraction; CN=Negative control; ns=not significant.

To identify any alterations in distinct cell cycle phases (G0-G1, S, and G2-M phases) of the parasites, cell cycle assay was performed with parasites challenged with fractions at the IC_50_ (Figures 4(a) and 4(b)). The assay was designed to capture the interaction between propidium iodide and DNA [29]. Overall, there was a significant reduction of G0-G1 phase from 61.43% in the negative control to 50.80% (*P*<0.001), 52.60% (*P*<0.01), and 55.25% (*P*<0.05) in ZRFD, ZRFM, and ZRWB, respectively (Figure 4(b), Additional file 3). Also, there was a significant increase of G2-M phase from 22.93% in the negative control to 31.48%, 29.18%, and 30.18% in ZRFD, ZRFM, and ZRWB, respectively (*P*<0.05) (Figure 4(b), Additional file 3). Moreover, ZRFD and ZRFM significantly increased the S phase from 15.53% in the untreated control to 19.08% and 19.03%, respectively (*P*<0.05) (Figure 4(b), Additional file 3).

### 3.3. *Z. zanthoxyloides *LZT (Root) Alters Morphology and Distribution of* T. brucei*

**Figure 5:**
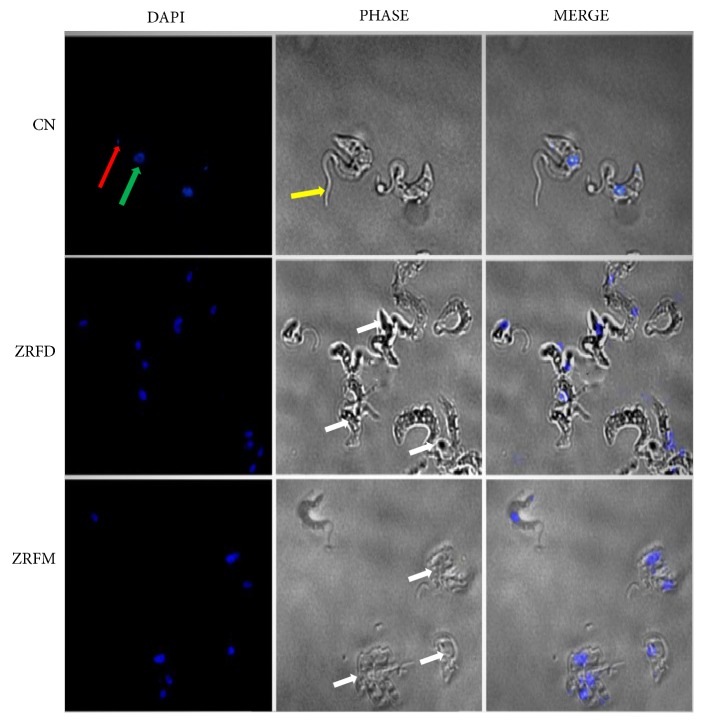
*Effect of selected fractions on cell morphology and structure of T. brucei. *Cells were mostly distorted or clustered as compared to the negative control (white arrows). Red arrow=kinetoplast; green arrow=nucleus; yellow arrow=flagellum; ZR=*Z. zanthoxyloides *LZT, root; FD=Dichloromethane fraction; FM=Methanol fraction; CN=Negative control.

Exploration of antitrypanosomal effects on parasite morphology can provide insights into intracellular mechanisms of antitrypanosomal action. Parasites challenged for 24 hours at the IC_50_ of the two most promising fractions (ZRFD and ZRFM) were observed for morphological changes via fluorescence microscopy using DAPI. ZRFD and ZRFM induced severe distortion of parasite morphology and resulted in the aggregation of a considerable number of parasites (Figure 5).

**Figure 6:**
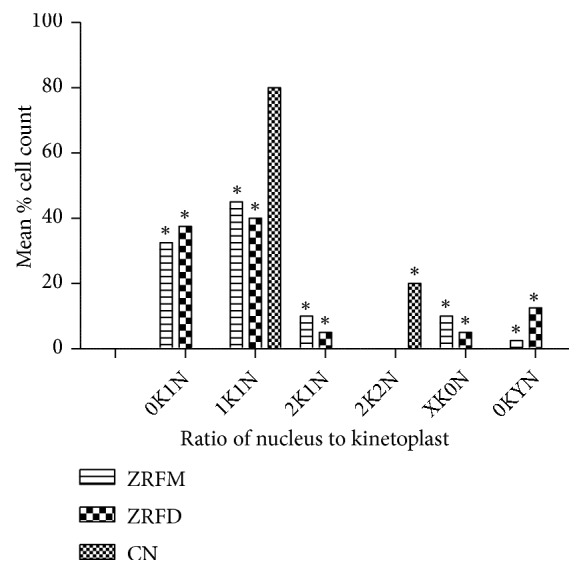
*Effect of selected fractions on nuclei and kinetoplasts in T. brucei*. Two hundred and forty cells were counted to determine effects of selected fractions on number of nuclei and kinetoplasts using fluorescence microscopy. P values were calculated from 3 distinct counts (*∗P *< 0.05). ZR=*Z. zanthoxyloides *LZT, root; FD=Dichloromethane fraction; FM=Methanol fraction; CN=Negative control. K=Kinetoplast; N=nucleus; X and Y=variables (X>2, Y>4).

The effect of fractions on parasite morphology was strengthened by the observed alterations in the number of nuclei and kinetoplasts. In comparison to the negative control, ZRFD and ZRFM variably altered the ratio of nuclei to kinetoplasts in distinct populations of the parasites (Figure 6). In the majority of affected parasites, ZRFD and ZRFM resulted in the respective loss of kinetoplasts in 37.5% and 32.5% of cells (*P*<0.01), while keeping the nuclei relatively intact (Figure 6). In yet another population of relatively few cells (ZRFD, 12.5%; ZRFM, 2.5%;* P*<0.05), the loss of kinetoplasts was four times more than the nuclei (Figure 6). Furthermore, the fractions resulted in a complete loss of the nuclei in other population of parasites while more than doubling the kinetoplasts (ZRFD, 5.0%; ZRFM, 10.0%;* P*<0.05) (Figure 6).

## 4. Discussion

Trypanosomiasis chemotherapy is beset with various challenges such as drug resistance, undesirable side effects, and difficulty in regimen application [5–10]. The medicinal values of* Zanthoxylum *may be attributed to the rich array of pharmacological and phytochemical properties [12, 31]. In view of the urgent need to circumvent the challenges associated with the currently available antitrypanosomal drugs, determination of the mechanisms of antitrypanosomal action of* Zanthoxylum *may facilitate drug discovery in AT. The present study is probably the first time the potential effects of* Z. zanthoxyloides *LZT on critical aspects of the cell biology of* T. brucei*, namely, cell cycle, cell morphology, and induction of cell death, are investigated.

The methanol, dichloromethane, and butanol fractions of* Z. zanthoxyloides *LZT (root) exhibited the most promising selective antitrypanosomal activities which may be attributed to the presence of secondary metabolites. The similarity in effects and mechanisms of antitrypanosomal actions observed for methanol and dichloromethane fractions may be partially accounted for by the presence of the same major secondary metabolites in the two fractions. Probably, major compounds that were not completely extracted by dichloromethane in the Kupchan solvent extraction ended up in the methanol fraction. Despite the similarities in their chromatographic and spectroscopic profiles, the methanol and dichloromethane fractions also differed in the absorption intensities and retention times of other minor but potentially active antitrypanosomal compounds which could have contributed to subtle but important differences in the observed antitrypanosomal activities between the two fractions. Furthermore, the dose-response curves of all fractions indicated inverted Hill coefficients less than -1, which might be an indication of positively cooperative binding [30]. This suggests that the interaction between compounds of the fractions and the parasite may increase the affinity of molecules of the same or other compounds towards the parasite thereby leading to a synergistic reduction of cell viability.

Distinct mechanisms of cell death have been observed in* Trypanosoma *[32–35]. The essential oil of* Zanthoxylum bungeanum *was also reported to induce apoptosis in HaCaT human keratinocytes [36]. However, the present study is the first time a species of* Zanthoxylum *is reported to induce apoptosis-like cell death in* T. brucei*. Dichloromethane, methanol, and butanol fractions of* Z. zanthoxyloides *LZT significantly induced apoptosis-like cell death in* T. brucei*. All fractions practically exerted no effect on necrosis-like cell death at the observed concentrations. However, the importance of other mechanisms of cell death not investigated in the present study cannot be ruled out [33, 37]. The observation of autophagy in* T. brucei *highlights the importance of other potential mechanisms of cell death in the parasite [33]. Studies that will consider the induction of other types of cell death by* Z. zanthoxyloides *LZT could be investigated. It is however important to interpret with caution apoptosis and necrosis mechanisms in unicellular protozoans since these protists do not encode caspases in their genome [37].

As other eukaryotes, the cell cycle of trypanosomes includes four phases: G1, S, G2, and M phases [38].* Zanthoxylum *has been reported to affect the cell cycle of different types of cells such as the human hepatoma-derived cell lines and human colon adenocarcinoma cell lines (Chou et al., 2011) [39]. However, the present study is the first time* Zanthoxylum *is shown to affect the cell cycle of trypanosomes. All the fractions significantly reduced the number of cells in the G0-G1 phase while increasing the G2-M population. This suggests that the fractions constrained most of the parasites to the G2-M phase thereby inhibiting karyokinesis and cytokinesis [38]. The complete separation of parasites into two daughter cells would therefore be inhibited, thereby relatively increasing the number of tetraploid cells at the G2-M phase. Moreover, all the fractions caused an increase in the S phase population of parasites, thereby suggesting an inhibitory effect on DNA synthesis.

The fractions also exerted morphological and structural changes on the parasites. ZRFD and ZRFM induced severe distortion and aggregation of the parasites. Interestingly, the fractions variably altered the ratio of kinetoplasts to nuclei in distinct populations of the parasites. However, in the majority of the parasites, this alteration resulted in the loss of kinetoplasts while keeping the nuclei relatively intact. The kinetoplast consists of circular DNA molecules that are topologically relaxed and interlocked to form a network of minicircles and maxicircles involved in RNA editing [40–42]. Moreover, the beginning and completion of kinetoplast DNA replication usually precedes that of the nuclear DNA in* T. brucei *[38]. By the end of the G2 phase, the replication of the kinetoplast is usually complete while that of the nucleus is about half-way through [38]. Therefore, it is possible that ZRFD and ZRFM may have impact on the nuclei and kinetoplasts through selective interactions with enzymes responsible for the synthesis of proteins and RNA during G1 and G2 phases. However, further studies are required to fully understand the apparent variation in effects between the nucleus and the kinetoplast.

## 5. Conclusions

In this study, the antitrypanosomal activity of* Z. zanthoxyloides *LZT (root) with regard to effects on the cell viability, cell death, cell cycle, morphology, and distribution of* T. brucei*, as well as the chromatographic and spectroscopic characterization of the antitrypanosomal compounds, was investigated.* Z. zanthoxyloides *LZT (root) was found to exhibit significant effects on apoptosis-like cell death with a remarkable alteration in the cell cycle of* T. brucei*. The antitrypanosomal compounds consisted mainly of aromatic hydrocarbons that could largely be responsible for the observed antitrypanosomal activities of the fractions. Our results suggest that* Z. zanthoxyloides *LZT holds significant potential for chemotherapeutic interventions in African trypanosomiasis. The identification and subsequent determination of the antitrypanosomal effects of these compounds would further deepen our understanding of the mechanisms of antitrypanosomal actions for the purpose of drug discovery in AT.

## Figures and Tables

**Table 1:** Effect of the crude extracts of *Z. zanthoxyloides *LZT on cell viability of *T. brucei, L. donovani, P. falciparum, *and Jurkat cells.

Part of plant used	MEAN IC_50_ ±SEM (*μ*g/ml)				SI		
	Jurkat	*T. brucei*	*L. donovani*	*P. falciparum*	*T. brucei*	*L. donovani*	*P. falciparum*
Root	247.16±0.05	3.41±0.03	45.2±0.10	334.77±0.02	72.48	12.91	0.74
Stem bark	583.53±0.02	39.43±0.04	13.5±0.04	112.15±0.01	6.27	18.30	5.20
Leaves	95.83±0.15	27.73±0.02	>1000±0.12	>1000±0.15	3.46	<0.10	<0.96

Mean IC_50_ and standard errors (SE) were calculated from three distinct experiments. Selectivity index (SI) was calculated as the ratio of IC_50_ in Jurkat to IC_50_ in the respective parasite.

**Table 2:** Wavenumbers for Kupchan fractions in IR spectroscopy.

*Peak*	*ZRC*		*ZRFH*		*ZRFD*		*ZRFM*		*ZRWB*	
	cm^−1^(x)	%T(y)	cm^−1^ (x)	%T(y)	cm^−1^(x)	%T(y)	cm^−1^ (x)	%T(y)	cm^−1^ (x)	%T(y)
1	3321.82	82.62	3300.1	97.04	3286.06	86.31	3288.03	87.51	3323.31	70.01
2	2926.06	76.00	2923.81	65.26	2957.50	77.74	2957.30	78.11	2932.86	77.69
3	1657.39	78.24	2853.79	75.37	2925.93	77.42	2925.30	77.12	1699.41	81.61
4	1612.15	78.01	1743.38	73.01	2870.81	82.94	1656.31	64.24	1599.87	69.61
5	1551.17	80.14	1659.16	87.74	1656.29	62.85	1611.20	62.78	1511.53	64.64
6	1490.74	79.82	1630.76	89.39	1610.77	60.59	1547.67	65.37	1456.86	65.25
7	1454.42	75.31	1546.12	90.56	1548.04	63.26	1503.32	63.18	1427.81	66.09
8	1370.11	78.47	1490.71	91.92	1503.44	63.58	1489.69	56.43	1278.27	50.51
9	1247.16	69.35	1461.89	81.18	1489.74	55.56	1464.74	67.80	1217.08	51.48
10	1085.23	74.73	1372.99	87.77	1465.26	68.46	1446.14	58.12	1028.45	36.32
11	1042.2	61.93	1246.00	79.57	1446.23	57.17	1368.08	75.18	807.57	62.80
12	997.68	72.87	1161.00	74.84	1355.27	74.85	1244.88	41.57	762.34	55.93
13	878.83	78.23	1099.30	83.94	1245.12	40.54	1158.03	71.22	529.58	46.65
14	518.64	70.23	1040.97	84.02	1157.73	72.27	1099.19	71.95	NA	NA
15	NA	NA	997.43	81.07	1099.77	74.73	1037.68	51.18	NA	NA
16	NA	NA	879.69	90.99	1037.85	53.97	995.45	59.91	NA	NA
17	NA	NA	809.39	92.92	996.90	59.87	930.99	68.02	NA	NA
18	NA	NA	721.97	84.02	930.52	68.63	851.92	78.81	NA	NA
19	NA	NA	424.87	92.98	851.27	77.50	808.79	69.94	NA	NA
20	NA	NA	NA	NA	808.25	68.49	744.67	80.02	NA	NA
21	NA	NA	NA	NA	596.94	73.00	672.73	80.05	NA	NA
22	NA	NA	NA	NA	517.94	69.61	596.95	78.49	NA	NA
23	NA	NA	NA	NA	424.60	75.60	518.25	77.31	NA	NA
24	NA	NA	NA	NA	NA	NA	425.62	82.08	NA	NA

Ordinate(x) = %transmittance; Abscissa (y) = wavenumber; ZR= *Z. zanthoxyloides *LZT (root); ZRC= crude extract of ZR; ZRFH= *Z. zanthoxyloides *hexane fraction; ZRFD= *Z. zanthoxyloides *dichloromethane fraction; ZRFM= *Z. zanthoxyloides *methanol fraction; ZRWB= *Z. zanthoxyloides *butanol fraction; NA= not applicable.

**Table 3:** Retention times for Kupchan fractions in HPLC.

Peak	(x) RT (min)	Width (min)	Area (mAU*∗*s)	(y) Height (mAU)	Area (%)
*ZRC*					
1	2.872	0.1155	61.05810	7.38034	1.7119
2	3.167	0.0989	240.89647	37.04975	6.7541
3	3.373	0.1350	270.91974	37.48990	7.5958
4	3.730	0.1115	2536.17334	337.99835	71.1074
5	4.328	0.1290	115.58913	13.35508	3.2408
6	4.621	0.1279	15.72073	1.79944	0.4408
7	5.301	0.1223	129.75060	15.88849	3.6379
8	5.654	0.1332	33.90483	3.83241	0.9506
9	14.339	0.1536	101.36829	9.34081	2.8421
10	14.693	0.2510	61.30116	3.50620	1.7187

*ZRFH *					
1	2.879	0.1101	32.03850	4.44205	1.4512
2	3.183	0.0999	105.94155	15.86805	4.7988
3	3.377	0.0904	60.97616	9.96817	2.7620
4	3.479	0.0849	35.27485	6.24909	1.5978
5	3.746	0.1102	1726.87097	233.63256	78.2214
6	4.336	0.1189	71.96671	9.13256	3.2598
7	5.318	0.1276	102.20798	11.97620	4.6297
8	14.451	0.1441	72.39410	7.21304	3.2792

*ZRFD*					
1	2.902	0.1293	36.92966	4.47213	1.4285
2	3.185	0.1008	440.68698	64.46280	17.0464
3	3.372	0.0900	94.99347	15.39953	3.6745
4	3.482	0.0933	56.08873	9.30652	2.1696
5	3.743	0.1033	1624.33984	232.98772	62.8316
6	3.927	0.0924	206.64229	32.40942	7.9932
7	4.335	0.1152	41.74596	5.58815	1.6148
8	5.304	0.1243	36.92151	4.52034	1.4282
9	14.357	0.1347	46.87906	5.07797	1.8133

*ZRFM *					
1	2.891	0.1349	63.85921	7.53434	1.6574
2	3.185	0.1042	525.79871	74.60051	13.6468
3	3.374	0.0883	109.30883	18.13418	2.8370
4	3.485	0.0934	75.00216	12.25811	1.9466
5	3.745	0.1082	2825.35791	386.82672	73.3303
6	4.336	0.1164	70.74014	9.34014	1.8360
7	5.306	0.1243	61.64240	7.46639	1.5999
8	14.459	0.1800	121.21284	9.81946	3.1460

*ZRWB *					
1	2.874	0.1237	35.44300	4.41159	3.1619
2	3.174	0.1107	41.06397	5.46061	3.6634
3	3.374	0.1285	43.36354	4.61984	3.8685
4	3.732	0.1124	830.25043	109.60705	74.0683
5	4.324	0.1217	36.01653	4.53515	3.2131
6	5.28 9	0.1306	53.58618	6.15428	4.7805
7	14.342	0.1273	48.23082	5.29576	4.3028
8	14.605	0.2202	32.97150	2.06582	2.9415

Ordinate(x) = absorbance intensity; Abscissa (y) = retention time; ZR=*Z. zanthoxyloides *LZT (root); ZRC=crude extract of ZR; ZRFH= *Z. zanthoxyloides *hexane fraction; ZRFD= *Z. zanthoxyloides *dichloromethane fraction; ZRFM= *Z. zanthoxyloides *methanol fraction; ZRWB= *Z. zanthoxyloides *butanol fraction; NA= not applicable.

**Table 4:** Effect of Kupchan fractions on cell viability of *T. brucei *and human cell lines.

FRACTIONS	MEAN IC_50_ ± SE (*μ*g/ml)			SI	
	*T. brucei*	Jurkat	Chang liver	Jurkat	Chang liver
ZRC	3.41**±**0.06	NA	NA	NA	NA
ZRFD	5.70 ± 0.03	52.04 ± 0.04	89.93 **± **0.03	9.13	15.78
ZRFH	13.26 ± 0.11	NA	NA	NA	NA
ZRFM	3.89 ± 0.06	68.15 ± 0.05	93.84 ± 0.03	17.52	24.12
ZRWB	4.02 ± 0.06	25.28 ± 0.05	375.58 ± 0.07	6.29	93.43
DA	0.54 ± 0.05	NA	39.72 ± 0.03	NA	73.56
DX	NA	0.27 **±** 0.28	145.22 ± 0.02	NA	NA

Mean IC_50_ and standard errors (SE) were calculated from three distinct experiments. SI was calculated as the ratio of IC_50_ in Jurkat or Chang liver cells to the IC_50_ in *T. brucei. *ZR=*Z. zanthoxyloides*, root; ZRC=crude extract of ZR; ZRFH= *Z. zanthoxyloides *hexane fraction; ZRFD= *Z. zanthoxyloides *Dichloromethane fraction; ZRFM= *Z. zanthoxyloides *methanol fraction; ZRWB= *Z. zanthoxyloides *butanol fraction; DA=Diminazene aceturate, (an antitrypanosomal drug); DX=Doxorubicin, (an antileukaemia drug); SI= selectivity index; NA=Not applicable (only ZRFD, ZRFM, and ZRWB were considered for further analysis due to their promising IC_50_ values against *T. brucei*).

## Data Availability

The data used to support the findings of this study are included within the article.

## Abbreviations

*Z. zanthoxyloides* LZT: * Zanthoxylum zanthoxyloides* (Lam.) Zepern. & Timler
HAT: Human African trypanosomiasis
AAT: African animal trypanosomiasis
HPLC: High-performance liquid chromatography
ATR-IR: Attenuated total reflectance infrared spectroscopy
ZR: * Z. zanthoxyloides* LZT (root)
FH: Hexane fraction of ZR
FD: Dichloromethane fraction of ZR
WB: Butanol fraction of ZR
FM: Methanol fraction of ZR.

## Supplementary Materials

Supplementary Materials
* Additional file 1: attenuated total reflectance infrared spectra of Kupchan fractions*. Mid-infrared spectroscopy was carried out by the Universal Attenuated Total Reflectance (UATR) spectrometer. Ordinate= transmittance intensity (%T); abscissa=wavenumber (cm-^1^); ZR=*Z. zanthoxyloides *LZT (root); ZRC=crude extract of ZR; FD=Dichloromethane fraction; FM=methanol fraction; WB= butanol fraction.* Additional file 2: HPLC chromatograms of Kupchan fraction.* HPLC was carried out at 254 nm using a gradient solvent system of absolute acetonitrile and 0.1% V/V formic acid. Ordinate= absorbance intensity (mAU); abscissa=retention time (mins); ZR=*Z. zanthoxyloides *LZT (root); ZRC=crude extract of ZR; FD=Dichloromethane fraction; FM=methanol fraction; WB= butanol fraction.* Additional file 3: effect of selected fractions on cell cycle of T. brucei.* Mean percentage cell count was calculated from the dot distributions and histograms of the cell cycle in 4 distinct counts. Standard error of the mean (SEM) was calculated from 4 distinct cell counts. ZR=*Z. zanthoxyloides *LZT, root; BP=*B. pilosa*, whole plant; WB=butanol fraction; FD=Dichloromethane fraction; FM=Methanol fraction; CN=Negative control.

## References

[1] Simarro P. P., Giuliano C., Franco J. R. (2012). Estimating and mapping the population at risk of sleeping sickness. *PLOS Neglected Tropical Diseases*.

[2] Morrison L. J., Vezza L., Rowan T., Hope J. C. (2016). Animal African trypanosomiasis: time to increase focus on clinically relevant parasite and host species. *Trends in Parasitology*.

[3] Cnops J., Magez S., De Trez C. (2015). Escape mechanisms of African trypanosomes: why trypanosomosis is keeping us awake. *Parasitology*.

[4] Steverding D. (2010). The development of drugs for treatment of sleeping sickness: a historical review. *Parasites & Vectors*.

[5] Melaku A., Birasa B. (2013). Drugs and drug resistance in african animal trypanosomosis: a review. *European Journal of Applied Sciences*.

[6] Scott A. G., Tait A., Turner C. M. R. (1996). Characterisation of cloned lines of Trypanosoma brucei expressing stable resistance to MelCy and suramin. *Acta Tropica*.

[7] Matovu E., Geiser F., Schneider V. (2001). Genetic variants of the TbAT1 adenosine transporter from African trypanosomes in relapse infections following melarsoprol therapy. *Molecular and Biochemical Parasitology*.

[8] Baker N., Alsford S., Horn D. (2011). Genome-wide RNAi screens in African trypanosomes identify the nifurtimox activator NTR and the eflornithine transporter AAT6. *Molecular and Biochemical Parasitology*.

[9] Barrett M. P., Vincent I. M., Burchmore R. J., Kazibwe A. J., Matovu E. (2011). Drug resistance in human African trypanosomiasis. *Future Microbiology*.

[10] Franco J., Simarro P., Diarra A., Ruiz-Postigo J., Samo M., Jannin G. (2012). Monitoring the use of nifurtimox-eflornithine combination therapy (NECT) in the treatment of second stage gambiense human African trypanosomiasis. *Research and Reports in Tropical Medicine*.

[11] Tagboto S., Townson S. (2001). Antiparasitic properties of medicinal plants and other naturally occurring products. *Advances in Parasitology*.

[12] Patino L. O. J., Prieto R. J. A., Cuca S. L. E. (2012). *Zanthoxylum*genus as potential source of bioactive compounds. *Bioactive Compounds in Phytomedicine*.

[13] Adesina S. K. (2005). The Nigerian *Zanthoxylum*; chemical and biological values. *African Journal of Traditional, Complementary and Alternative Medicine*.

[14] Prempeh A., Mensah-Attipoe J. (2008). Analgesic activity of crude aqueous extract of the root bark of zanthoxylum xanthoxyloides. *Ghana Medical Journal*.

[15] Azando E., Hounzangbé–Adoté M., Olounladé P. (2011). Involvement of tannins and flavonoids in the in vitro effects of Newbouldia laevis and Zanthoxylum zanthoxyloïdes extracts on the exsheathment of third-stage infective larvae of gastrointestinal nematodes. *Veterinary Parasitology*.

[16] Barnabas B. B., Mann A., Ogunrinola T. S., Anyanwu P. E. (2010). Screening for anthelminthic activities from extracts of Zanthoxylum zanthoxyloides, Neocarya macrophylla and Celosia laxa against ascaris infection in rabbits. *International Journal of Applied Research in Natural Products*.

[17] Ferreira M., Rojas de Arias A., Torres de Ortiz S. (2002). Leishmanicidal activity of two canthin-6-one alkaloids, two major constituents of Zanthoxylum chiloperone var. angustifolium. *Journal of Ethnopharmacology*.

[18] Ferreira M. E., Nakayama H., de Arias A. R. (2007). Effects of canthin-6-one alkaloids from Zanthoxylum chiloperone on Trypanosoma cruzi-infected mice. *Journal of Ethnopharmacology*.

[19] Ferreira M. E., Cebrián-Torrejón G., Corrales A. S. (2011). Zanthoxylum chiloperone leaves extract: first sustainable chagas disease treatment. *Journal of Ethnopharmacology*.

[20] Sen R., Chatterjee M. (2011). Plant derived therapeutics for the treatment of Leishmaniasis. *Phytomedicine*.

[21] Ngassoum M., Essia-Ngang J., Tatsadjieu L., Jirovetz L., Buchbauer G., Adjoudji O. (2003). Antimicrobial study of essential oils of Ocimum gratissimum leaves and Zanthoxylum xanthoxyloides fruits from Cameroon. *Fitoterapia*.

[22] Ouattara B., Angenot L., Guissou P. (2004). LC/MS/NMR analysis of isomeric divanilloylquinic acids from the root bark of Fagara zanthoxyloides Lam. *Phytochemistry*.

[23] Wouatsa V. N. A., Misra L., Kumar S. (2013). Aromatase and glycosyl transferase inhibiting acridone alkaloids from fruits of Cameroonian Zanthoxylum species. *Chemistry Central Journal*.

[24] Mann A., Ifarajimi O. R., Adewoye A. T. (2011). In vivo antitrypanosomal effects of some Ethnomedicinal plants from Nupeland of north central Nigeria. *African Journal of Traditional, Complementary and Alternative Medicines*.

[25] Mann A., O. Ogbadoyi E. (2012). Evaluation of medicinal plants from nupeland for their in vivo antitrypanosomal activity. *American Journal of Biochemistry*.

[26] Kupchan S. M., Britton R. W., Ziegler M. F., Sigel C. W. (1973). Bruceantin, a new potent antileukemic simaroubolide from Brucea antidysenterica. *The Journal of Organic Chemistry*.

[27] Vega-Avila E., Pugsley M. K. (2011). An overview of colorimetric assay methods used to assess survival or proliferation of mammalian cells. *Proceedings of the Western Pharmacology Society*.

[28] Wlodkowic D., Skommer J., Darzynkiewicz Z. (2009). Flow cytometry-based apoptosis detection. *Methods in Molecular Biology (Clifton, N.J.)*.

[29] Pozarowski P., Darzynkiewicz Z. (2004). Analysis of cell cycle by flow cytometry. *Methods in Molecular Biology*.

[30] Prinz H. (2010). Hill coefficients, dose–response curves and allosteric mechanisms. *Journal of Chemical Biology*.

[31] Medhi K., Deka M., Bhau B. S. (2013). The Genus *Zanthoxylum*- a stockpile of biological and ethnomedicinal properties. *Scientific Reports*.

[32] Welburn S. C., Dale C., Ellis D., Beecroft R., Pearson T. W. (1996). Apoptosis in procyclic Trypanosoma brucei rhodesiense in vitro. *Cell Death & Differentiation*.

[33] Schmidt R. S., Bütikofer P. (2014). Autophagy in trypanosoma brucei: amino acid requirement and regulation during different growth phases. *PLoS ONE*.

[34] de Silva Rodrigues J. H., Stein J., Strauss M. (2016). Clomipramine kills Trypanosoma brucei by apoptosis. *International Journal of Medical Microbiology*.

[35] Sousa P. L., Souza R. O., Tessarolo L. D. (2017). Betulinic acid induces cell death by necrosis in *Trypanosoma cruzi*. *Acta Tropica*.

[36] Li K., Zhou R., Jia W. W. (2016). *Zanthoxylum bungeanum* essential oil induces apoptosis of HaCaT human keratinocytes. *Journal of Ethnopharmacology*.

[37] Kaczanowski S., Sajid M., Reece S. E. (2011). Evolution of apoptosis-like programmed cell death in unicellular protozoan parasites. *Parasites & Vectors*.

[38] Vaughan S., Gull K. (2008). The structural mechanics of cell division in *Trypanosoma brucei*. *Biochemical Society Transactions*.

[39] Dung T. D., Chang H. C., Binh T. V. (2012). Zanthoxylum avicennae extracts inhibit cell proliferation through protein phosphatase 2A activation in HA22T human hepatocellular carcinoma cells in vitro and in vivo. *International Journal of Molecular Medicine*.

[40] Stuart K., Panigrahi A. K. (2002). RNA editing: complexity and complications. *Molecular Microbiology*.

[41] Liu Y., Englund P. T. (2007). The rotational dynamics of kinetoplast DNA replication. *Molecular Microbiology*.

[42] Schneider A., Bursać D., Lithgow T. (2008). The direct route: a simplified pathway for protein import into the mitochondrion of trypanosomes. *Trends in Cell Biology*.

