# Supplementary figures and images for: Antitrypanosomal Effects of Zanthoxylum zanthoxyloides (Lam.) Zepern. & Timler Extracts on African Trypanosomes
Source: Evid Based Complement Alternat Med. 2019 Jul 4;2019:1730452. doi: 10.1155/2019/1730452 (PMC6637693; doi:10.1155/2019/1730452)

**1.**


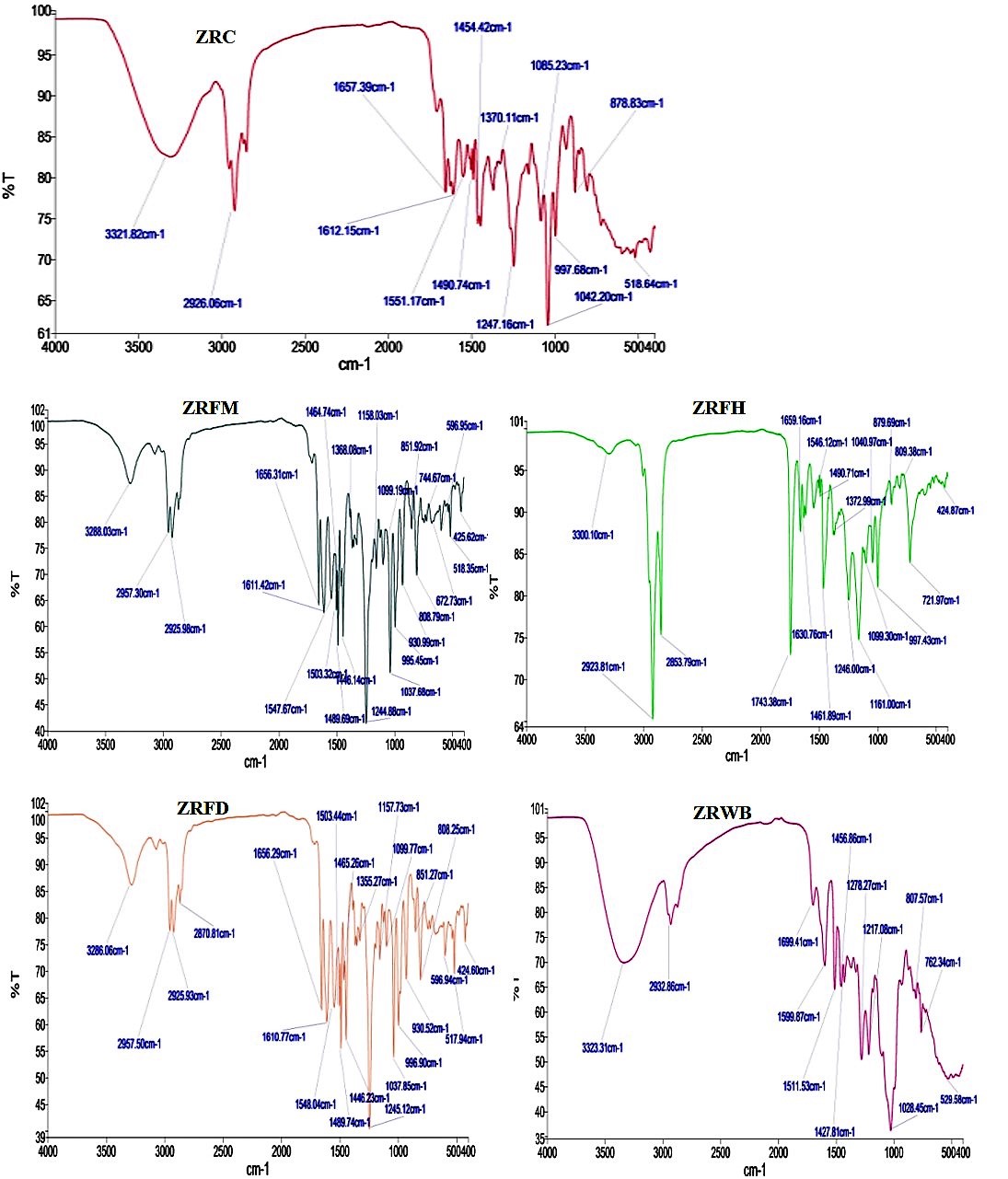


**2.**

**3.**

| **FRACTIONS** | **PERCENTAGE CELL COUNT (MEAN±SEM)** | | |
| --- | --- | --- | --- |
| **G0-G1 Phase** | **S Phase** | **G2-M Phase** |
| CN | 61.43 ± 1.26 | 15.53 **±** 1.25 | 22.93 ± 2.06 |
| ZRFD | 50.80 ±0.82 | 19.08 **±** 0.63 | 31.48 ± 1.20 |
| ZRFM | 52.60 ± 1.52 | 19.03 ± 0.71 | 29.18 ± 1.08 |
| ZRWB | 55.25 ± 1.12 | 16.10 ± 0.18 | 30.18 ± 1.87 |

Supplement: Supplementary Materials — Additional file 1: attenuated total reflectance infrared spectra of Kupchan fractions. Mid-infrared spectroscopy was carried out by the Universal Attenuated Total Reflectance (UATR) spectrometer. Ordinate= transmittance intensity (%T); abscissa=wavenumber (cm-1); ZR=Z. zanthoxyloides LZT (root); ZRC=crude extract of ZR; FD=Dichloromethane fraction; FM=methanol fraction; WB= butanol fraction. Additional file 2: HPLC chromatograms of Kupchan fraction. HPLC was carried out at 254 nm using a gradient solvent system of absolute acetonitrile and 0.1% V/V formic acid. Ordinate= absorbance intensity (mAU); abscissa=retention time (mins); ZR=Z. zanthoxyloides LZT (root); ZRC=crude extract of ZR; FD=Dichloromethane fraction; FM=methanol fraction; WB= butanol fraction. Additional file 3: effect of selected fractions on cell cycle of T. brucei. Mean percentage cell count was calculated from the dot distributions and histograms of the cell cycle in 4 distinct counts. Standard error of the mean (SEM) was calculated from 4 distinct cell counts. ZR=Z. zanthoxyloides LZT, root; BP=B. pilosa, whole plant; WB=butanol fraction; FD=Dichloromethane fraction; FM=Methanol fraction; CN=Negative control. [file 1730452.f1.docx]
